# Supplementary material for: Scaling and quantization of large-scale foundation model enables resource-efficient predictions in network biology
Source: Nat Comput Sci. 2026 Mar 27;6(5):450–63. doi: 10.1038/s43588-026-00972-4 (PMC13215881; doi:10.1038/s43588-026-00972-4)
Supplement: Supplementary file 1 — Reporting Summary [file 43588_2026_972_MOESM1_ESM.pdf]

nature portfolio

Corresponding author(s): Christina Theodoris

Last updated by author(s): Jun 26, 2025

Reporting Summary

Nature Portfolio wishes to improve the reproducibility of the work that we publish. This form provides structure for consistency and transparency in reporting. For further information on Nature Portfolio policies, see our Editorial Policies and the Editorial Policy Checklist.

Statistics

For all statistical analyses, confirm that the following items are present in the figure legend, table legend, main text, or Methods section.

n/a	Confirmed	
☐	☒	The exact sample size (*n*) for each experimental group/condition, given as a discrete number and unit of measurement
☐	☒	A statement on whether measurements were taken from distinct samples or whether the same sample was measured repeatedly
☐	☒	The statistical test(s) used AND whether they are one- or two-sided<br>*Only common tests should be described solely by name; describe more complex techniques in the Methods section.*
☐	☒	A description of all covariates tested
☐	☒	A description of any assumptions or corrections, such as tests of normality and adjustment for multiple comparisons
☐	☒	A full description of the statistical parameters including central tendency (e.g. means) or other basic estimates (e.g. regression coefficient) AND variation (e.g. standard deviation) or associated estimates of uncertainty (e.g. confidence intervals)
☐	☒	For null hypothesis testing, the test statistic (e.g. *F*, *t*, *r*) with confidence intervals, effect sizes, degrees of freedom and *P* value noted<br>*Give P values as exact values whenever suitable.*
☒	☐	For Bayesian analysis, information on the choice of priors and Markov chain Monte Carlo settings
☒	☐	For hierarchical and complex designs, identification of the appropriate level for tests and full reporting of outcomes
☐	☒	Estimates of effect sizes (e.g. Cohen's *d*, Pearson's *r*), indicating how they were calculated

Our web collection on statistics for biologists contains articles on many of the points above.

Software and code

Policy information about availability of computer code

Data collection

mygene https://github.com/biothings/mygene.py
requests https://github.com/psf/requests

Data analysis

The Geneformer models and related code are available on Hugging Face Model Hub (https://huggingface.co/ctheodoris/Geneformer).

Other software used in this study:
anndata https://github.com/scverse/anndata 0.10.7
CellRanger https://www.10xgenomics.com/support/software/cell-ranger/latest 2.2, 3, 7
cudatoolkit https://github.com/Jimver/cuda-toolkit 11.8.0
datasets https://github.com/huggingface/datasets 2.19.2
deepspeed https://github.com/microsoft/DeepSpeed 0.15.3
hyperopt https://github.com/hyperopt/hyperopt 0.2.7
LoomExperiment https://bioconductor.org/packages/release/bioc/html/LoomExperiment.html 1.8.0
loompy https://github.com/linnarsson-lab/loompy 3.0.7
matplotlib https://github.com/matplotlib/matplotlib 3.9.0
multiprocess https://github.com/uqfoundation/multiprocess 0.70.16
nccl https://github.com/NVIDIA/nccl 2.21.5.1
numpy https://github.com/numpy/numpy 1.26.4
optuna https://github.com/optuna/optuna 4.0.0
optuna-integration https://github.com/optuna/optuna-integration 4.0.0
peft https://github.com/huggingface/peft 0.12.0

pandas https://github.com/huggingface/peft 2.2.2
pyarrow https://github.com/apache/arrow 15.0.0
python https://www.python.org/ 3.10.13
pytz https://github.com/stub42/pytz 2024.1
pytorch https://github.com/pytorch/pytorch 2.3.1
ray https://github.com/ray-project/ray 2.24.0
scanpy https://github.com/scverse/scanpy 1.10.1
scikit-learn https://github.com/scikit-learn/scikit-learn 1.5.0
scipy https://github.com/scipy/scipy 1.13.1
seaborn https://github.com/scipy/scipy 0.13.2
setuptools https://github.com/pypa/setuptools 69.5.1
STAR https://github.com/alexdobin/STAR 2.7.8a
statsmodels https://github.com/statsmodels/statsmodels 0.14.2
tdigest https://github.com/tdunning/t-digest 0.5.2.2
tokenizers https://github.com/huggingface/tokenizers 0.21.0
tqdm https://github.com/tqdm/tqdm 4.66.4
transformers https://github.com/huggingface/transformers 4.48.2

For manuscripts utilizing custom algorithms or software that are central to the research but not yet described in published literature, software must be made available to editors and reviewers. We strongly encourage code deposition in a community repository (e.g. GitHub). See the Nature Portfolio guidelines for submitting code & software for further information.

Data

Policy information about availability of data

All manuscripts must include a data availability statement. This statement should provide the following information, where applicable:

- Accession codes, unique identifiers, or web links for publicly available datasets
- A description of any restrictions on data availability
- For clinical datasets or third party data, please ensure that the statement adheres to our policy

Genecorpus-104M is available on Hugging Face Dataset Hub at https://huggingface.co/datasets/theodoris-lab/Genecorpus-104M.

Research involving human participants, their data, or biological material

Policy information about studies with human participants or human data. See also policy information about sex, gender (identity/presentation), and sexual orientation and race, ethnicity and racism.

Reporting on sex and gender	N/A this study does not involve human research participants. All data used in this study was assembled from publicly available sources with original studies reporting relevant metadata characteristics.
Reporting on race, ethnicity, or other socially relevant groupings	N/A this study does not involve human research participants. All data used in this study was assembled from publicly available sources with original studies reporting relevant metadata characteristics.
Population characteristics	N/A this study does not involve human research participants. All data used in this study was assembled from publicly available sources with original studies reporting relevant metadata characteristics.
Recruitment	N/A this study does not involve human research participants. All data used in this study was assembled from publicly available sources with original studies reporting relevant study details.
Ethics oversight	N/A this study does not involve human research participants. All data used in this study was assembled from publicly available sources with original studies reporting relevant study details.

Note that full information on the approval of the study protocol must also be provided in the manuscript.

Field-specific reporting

Please select the one below that is the best fit for your research. If you are not sure, read the appropriate sections before making your selection.

☒ Life sciences ☐ Behavioural & social sciences ☐ Ecological, evolutionary & environmental sciences

For a reference copy of the document with all sections, see nature.com/documents/nr-reporting-summary-flat.pdf

Life sciences study design

All studies must disclose on these points even when the disclosure is negative.

Sample size	For the pretraining, we collected ~104 million human single cell transcriptomes from 2,903 publicly available datasets contributing towards a breadth of represented human tissues. These were all datasets we had access to in the public domain at the time of the study and were sufficient based on downstream task evaluations to gain meaningful biological knowledge during the pretraining.

Data exclusions

For the assembly of Genecorpus-104M, human single-cell transcriptomes were gathered from publicly available datasets with cross-referenced DOIs to ensure unique datasets and avoid the inclusion of duplicated cells. Raw and unfiltered data files were processed to remove empty droplets and debris using STAR with CellRanger 2.2 (run mode -soloCellFiltered). Datasets were additionally filtered to retain cells that contained a minimum of seven detected Ensembl-annotated protein-coding genes.

Replication

We separated data into training, validation, and test sets for all zero-shot and fine-tuning experiments. Results were replicated across different seeds for the model and different trials for timing; all replication attempts were successful.

Randomization

Data was split randomly into training, validation, and test sets for all zero-shot and fine-tuning experiments, either as individual genes for gene classification experiments where the test set contained held-out genes, or as individual cells for cell classification experiments, where the test set contained held-out cells.

Blinding

Blinding was not relevant to our study; all results were quantitative and deterministic model outputs.

Reporting for specific materials, systems and methods

We require information from authors about some types of materials, experimental systems and methods used in many studies. Here, indicate whether each material, system or method listed is relevant to your study. If you are not sure if a list item applies to your research, read the appropriate section before selecting a response.

Materials & experimental systems

n/a	Involved in the study
☒	☐ Antibodies
☒	☐ Eukaryotic cell lines
☒	☐ Palaeontology and archaeology
☒	☐ Animals and other organisms
☒	☐ Clinical data
☒	☐ Dual use research of concern
☒	☐ Plants

Methods

n/a	Involved in the study
☒	☐ ChIP-seq
☒	☐ Flow cytometry
☒	☐ MRI-based neuroimaging

Plants

Seed stocks

Report on the source of all seed stocks or other plant material used. If applicable, state the seed stock centre and catalogue number. If plant specimens were collected from the field, describe the collection location, date and sampling procedures.

Novel plant genotypes

Describe the methods by which all novel plant genotypes were produced. This includes those generated by transgenic approaches, gene editing, chemical/radiation-based mutagenesis and hybridization. For transgenic lines, describe the transformation method, the number of independent lines analyzed and the generation upon which experiments were performed. For gene-edited lines, describe the editor used, the endogenous sequence targeted for editing, the targeting guide RNA sequence (if applicable) and how the editor was applied.

Authentication

Describe any authentication procedures for each seed stock used or novel genotype generated. Describe any experiments used to assess the effect of a mutation and, where applicable, how potential secondary effects (e.g. second site T-DNA insertions, mosiacism, off-target gene editing) were examined.
